# Text mining tweets on e-cigarette risks and benefits using machine learning following a vaping related lung injury outbreak in the USA

**DOI:** 10.1016/j.health.2022.100066

**Published:** 2022-11

**Authors:** Lamiece Hassan, Mohab Elkaref, Geeth de Mel, Ilze Bogdanovica, Goran Nenadic

**Affiliations:** a Division of Informatics, Imaging and Data Sciences, The University of Manchester, UK; b IBM Research, Daresbury, UK; c School of Medicine, University of Nottingham, UK; d School of Computer Science, The University of Manchester, UK

**Keywords:** ENDS, Electronic nicotine delivery systems, EVALI, E-cigarette or Vaping product use Associated Lung Injury, THC, tetrahydrocannabinol, Social media, e-cigarettes, ENDS, EVALI, Public health, Machine learning, Twitter, UK, USA

## Abstract

Electronic nicotine delivery systems (ENDS) (also known as ‘e-cigarettes’) can support smoking cessation, although the long-term health impacts are not yet known. In 2019, a cluster of lung injury cases in the USA emerged that were ostensibly associated with ENDS use. Subsequent investigations revealed a link with vitamin E acetate, an additive used in some ENDS liquid products containing tetrahydrocannabinol (THC). This became known as the EVALI (E-cigarette or Vaping product use Associated Lung Injury) outbreak. While few cases were reported in the UK, the EVALI outbreak intensified attention on ENDS in general worldwide. We aimed to describe and explore public commentary and discussion on Twitter immediately before, during and following the peak of the EVALI outbreak using text mining techniques. Specifically, topic modelling, operationalised using Latent Dirichlet Allocation (LDA) models, was used to discern discussion topics in 189,658 tweets about ENDS (collected April–December 2019). Individual tweets and Twitter users were assigned to their dominant topics and countries respectively to enable international comparisons. A 10-topic LDA model fit the data best. We organised the ten topics into three broad themes for the purposes of reporting: informal vaping discussion; vaping policy discussion and EVALI news; and vaping commerce. Following EVALI, there were signs that informal vaping discussion topics decreased while discussion topics about vaping policy and the relative health risks and benefits of ENDS increased, not limited to THC products. Though subsequently attributed to THC products, the EVALI outbreak disrupted online public discourses about ENDS generally, amplifying health and policy commentary. There was a relatively stronger presence of commercially oriented tweets among UK Twitter users compared to USA users.

## Highlights

• In 2019 there was an e-cigarete related lung injury outbreak in the USA.
• The outbreak prompted discussions among Twiter users about e-cigarete benefits and risks.
• We analysed 189,658 tweets about e-cigaretes using machine learning models.
• Topics of tweets addressed vaping policy, sales, and the relative health risks and benefits.
• The lung injury outbreak may have diversified online discourses about e-cigaretes.

## 1 Introduction

Electronic nicotine delivery systems (ENDS) (also known as ‘e-cigarettes’) have transformed the tobacco product market over last decade. Many people claim that ENDS have helped them to stop smoking tobacco, however the evidence base on long-term health impacts is still developing [1]. Studies of ENDS have demonstrated effects on smoking cessation [1]; Nonetheless, concerns still persist about the safety of e-cigarettes [2] and the potential for e-cigarettes to be used by those – particularly younger people – with no prior use of tobacco (so called ‘never smokers’) or for other reasons aside from some smoking cessation. Indeed, surveys indicate that curiosity, social reasons and enjoyment of flavours were all common reasons for use among younger populations [3], [4].

The UK has been described as a global outlier with respect to its stance on ENDS [5]. In the UK, as with many countries internationally, ENDS are now subject to formal regulations governing their sale, packaging and accessibility [6]. Two major systematic reviews at the end of 2018 – from the UK [7] and the USA [8] – reviewed the same evidence about ENDS, yet came to very different conclusions about suggestions for public health policy [5]. The UK review went as far to assert that vaping is “at least 95% less harmful than smoking” [7], although this claim has been contested [9]. While other countries have been more cautious in their approaches towards ENDS, [10] Public Health England has since, under the banner of harm reduction, issued guidance to health professionals to advise smokers on using ENDS as a smoking cessation intervention [11]. Meanwhile, the USA has passed restrictions on ENDS with the intent of discouraging use among youths, although the effects remain unclear [12].

In August 2019, worldwide attention to ENDS harms and policy intensified following the sudden emergence of a cluster of lung injury cases in the USA dubbed the ‘EVALI’ (E-cigarette or Vaping product use Associated Lung Injury) outbreak. EVALI, which peaked in September 2019, affected mainly men and those aged 35 years and under [13]. By February 2020 it had resulted in 2807 hospitalisations and 68 deaths in the USA [14]. Two potential cases were also been identified in the UK [15]. Although initially the causes were unclear, subsequent investigations revealed that EVALI cases were strongly associated with vitamin E acetate, an additive used as a thickening (cutting) agent in some ENDS liquid products containing tetrahydrocannabinol (THC) [16], [17].

Although licit products on the UK market do not contain THC or vitamin E acetate [18], negative media coverage conflating different e-cigarette products – including the term ‘EVALI’ itself – may have affected attitudes among the UK general public about vaping in general. Previous studies have identified social media as an important battleground for shaping the debate around ENDS, using data-driven approaches to yield valuable insights into how ENDS are marketed, discussed and perceived [19], [20], [21], [22], [23], [24].

In this study we aimed to use text mining approaches to describe and explore public commentary and discussion on Twitter immediately before, during and following the peak of the EVALI outbreak. Like many other social media platforms, Twitter [25] allows members to post messages, known as ‘tweets’, and interact with each other in various ways, for example by sharing, subscribing to, and commenting on each other’s’ posts. What perhaps differentiates Twitter from other popular platforms is the focus on brevity – tweets are limited to 280 characters – and its highly public nature. Twitter is popular with news outlets, celebrities and politicians globally. One recent study found that adults who used Twitter in the USA were comparatively younger, more highly educated and had higher incomes than the general public [26]. Though Twitter members can choose to apply privacy restrictions, most tweets are publicly accessible and viewable even without registering with the platform. Such qualities have made Twitter a rich resource and a popular platform for conducting ENDS research [19], [20], [21], [22], [23], [24].

Specifically, in this study we aimed to (a) determine the nature and relative prevalence of topics of discussion online about ENDS and vaping and (b) identifying geographic differences in tweet topics, particularly between people who use Twitter in the UK and USA.

**Fig. 1:**
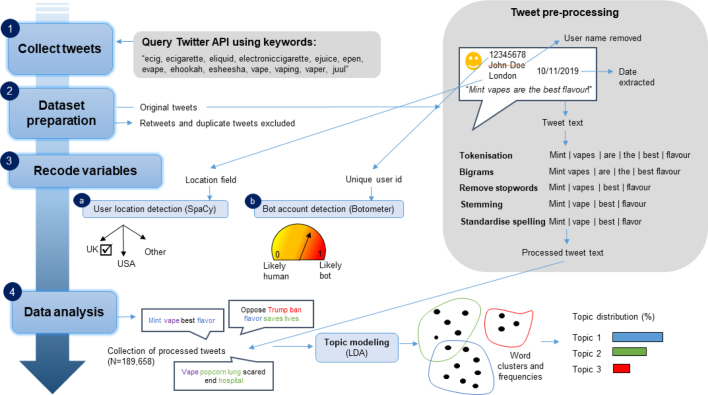
Tweet analysis pipeline overview. Legend: Overview of text data collection and analysis pipeline. Summary version of keywords presented for brevity. The full combination of keywords used to query the API included plural versions (e.g. ecigs) and prefix variations (e.g. e-cig and ecig).(For interpretation of the references to colour in this figure legend, the reader is referred to the web version of this article.)

## 2 Methods

This study used Twitter data [27]. Fig. 1 provides an overview of the key phases used to collect, prepare, recode and analyse Twitter data as part of this study, with illustrations of the key processes and methods. Each of these phases are described in turn in the following paragraphs. Data were collected, processed and analysed in Python (version 3.6) unless otherwise specified.

### 2.1 Data collection

We collected tweets about ENDS using Twitter’s application programming interface (API version 2) under the academic research product track [25]. To extract a sample of relevant tweets, we queried the API using ENDS related keywords, which were tested and refined in an iterative process, described as follows. First, we identified commonly used keywords about ENDS used to search Twitter in the previous literature [20], [28]. Then we tested varying combinations of these keywords and reviewed convenience samples of the results to judge their relevance to ENDS. To help choose between different options, batches of tweets (N = 100) generated using keyword searches were manually reviewed and classified (relevant or non-relevant). Including the names of common e-cigarette brands (e.g. ‘Blu’) as keywords yielded a higher proportion (>50%) of non-relevant tweets not specific to e-cigarettes (e.g. the colour ‘blue’). The only exception to this was ‘JUUL’ (the name of the leading brand in the USA); thus this particular brand keyword was retained. Tweets containing only ‘vapour’ related terms (‘vapour’, ‘vapouring’) also yielded low relevance, due to the overlap with weather related tweets (<15% were relevant to ENDS).

The final combination of keywords (Fig. 1) included singular and plural terms about e-cigarettes and vaping, using both USA and UK spelling variations. We used this to collect a sample of tweets that met the following inclusion criteria: includes one or more pre-defined keywords; posted between 1st April and 31st December 2019; and user language set to English. Rate limits and data limits imposed by Twitter’s terms and conditions and practical considerations (i.e. time, storage and computing power) prevented collection and analysis of all available tweets. Rather, we aimed to sample 1000 tweets per day throughout the observation period. This sample size was chosen to pragmatically balance performance-based and practical considerations in light of our chosen data analysis methods. Due to the limits of the API, sampling was not strictly random, although efforts were made to sample tweets from different times of day. The observation period was chosen to capture the period before, during and after the peak of EVALI—related hospital admissions in September 2019.

### 2.2 Dataset preparation

Duplicate tweets were removed. The textual content of all tweets collectively (known as the ‘corpus’) was retained for analysis, plus a limited amount of metadata (including date and geo-coordinate data) and members (including user identification number and location).

Tweet text was prepared for analysis using standard natural language processing techniques [29], [30], [31]. This included: removing special characters (e.g. hashtags and emojis); separating out words, known as ‘tokenisation’; forming two-word combinations of adjacent words, known as ‘bigrams’; stripping suffixes, known as ‘stemming’ [32] (e.g. ‘smokes’, ‘smoking’ and ‘smoked’ become ‘smoke’); and removing uninformative words (e.g. ‘of’, ‘the’, ‘and’). Slang (non-standard) language was left intact; however, we did manually review the most frequently appearing 200 words and normalised alternative USA and UK spellings of the same words into the same spelling (e.g. ‘flavor’ and ‘flavour’ both became ‘flavor’). A ‘bag-of-words’ representation – so-called because it ignores word order and grammatical structure – was used to represent the occurrence of words and bigrams and their frequencies within tweets.

As expected, less than 1% (N = 94) of tweets included precise geographical coordinates. Country-specific searches were possible using the API, but also yielded comparatively low numbers of tweets during testing. Instead, we used the location field, completed by Twitter members using free text, to infer the locations of members and derive datasets of UK and USA members for comparison. Named entity recognition tools (using the spaCy library [30]) were used to automatically identify geographical locations mentioned in the location field; to boost this matching process, we added lists of major locations in the UK (defined as cities and towns with over 75,000 people [33]) and USA (defined as principal cities with over 50,000 people [34], plus all state names). Members were subsequently assigned to at least one of the following four location categories: the USA, the UK, neither and not known. Mentions of ambiguous locations that could be assigned to either the UK or USA (e.g. ‘Manchester’ and ‘Washington’) were reviewed and resolved manually by LH (N = 152). Members who indicated locations relevant to the USA and the UK (e.g. ‘New York/London’) were assigned to both location categories. The ‘not known’ category was used in cases where there was insufficient information to make a decision. Unique member numbers were used to link tweets with members. To estimate the accuracy of our algorithmic methods, a random sample of 100 tweet locations were reviewed manually by a researcher (LH) to determine whether human judgement matched the locations automatically derived via our combination of methods (UK; USA; Other; None). Of the 100 locations, human and algorithmically derived locations matched perfectly in 70 cases; of the non-matches, in 28/30 cases the algorithm failed to pick up a USA location identified by the researcher.

We used the BotometerLite API [35] to assess the likelihood that Twitter accounts were partially or fully controlled by software (or ‘bots’ i.e. robots). BotometerLite was used to generate a bot score of 0 to 1 for each account (higher scores indicating a higher likelihood of automated bot activity) and was selected as it is lightweight and capable of efficiently analysing accounts in bulk. All tweets were retained for analysis regardless of Botometer score.

### 2.3 Data analysis

Descriptive statistics (counts and percentages) were used to characterise members, tweets and the frequencies of common words and bigrams. A Sankey plot was used to visualise relative word frequency in the UK and USA using R (version 1.2.1335).

Topic modelling was used to automatically infer the presence of topics within the corpus using Latent Dirichlet Allocation (LDA), an unsupervised machine learning method regarded as a standard topic modelling method suitable for large corpora [36]. Briefly, LDA is a generative probabilistic model: it seeks to convert unordered, bag-of-words representations of documents (in this case tweets) and to represent each individual document as a probability distribution over a given number of topics [37]. LDA generates sets of statistically associated words know as ‘word sets’ that are proposed to theoretically signify latent topics within documents and across corpora. Crucially, unlike classical clustering models, LDA treats documents as a mixture of a fixed number of topics and so under LDA, each document in a corpus can be associated with multiple topics.

Using the ‘gensim’ library in Python [38], we tested LDA models over our bag-of-words corpus of tweets, varying fixed parameters including number of topics, passes and iterations. To evaluate different models and choose the optimal model, we used a combination of statistical methods, visualisations and manual inspection. First, we calculated and plotted intrinsic statistical measures of topic coherence including $C_{V}$ scores, which is a topic quality measure that quantifies and aggregates associations between words included in topic word sets and correlates well with human judgements [39]. Higher scores indicate higher topic coherence. Second, we shortlisted models with higher topic coherence, manually inspected word sets and produced visualisations to understand the content, size, strength and relationships of topics. This allowed us to assess which model offered the optimal balance between topic coherence and human interpretability. Using the final model, we estimated the contribution (%) of each topic present in each tweet and assigned tweets to their dominant topic i.e. the topic with the highest proportion. We then read samples of the tweets most representative of each topic (>80%) to infer topic content and produced visualisations indicating intertopic distance i.e. the relationships between topics [40]. Topics were then labelled with names and brief textual descriptions reflecting our subjective interpretation of their meaning. We further took the step of grouping similar topics into broader themes to provide an organised structure to our reporting and aid interpretation of results. Deriving these thematic groupings involved subjectively judging the relatedness of topics based on the semantic similarity of word sets and sample tweets, and reviewing visualisations of intertopic distance.

Member locations were cross-tabulated against the distribution of dominant topics among tweets to infer how topics varied between the UK and USA. Chi squared tests were used to compare between-group differences in proportions for categorical data (*P* < .05). Due to skewed data, we calculated median (rather than mean) bot scores between categories of members and topics; differences were tested using Mann–Whitney U tests (*P* < .05).

### 2.4 Ethical considerations

This project used publicly available data and was at the time deemed exempt from formal ethical review by University of Manchester Research Ethics Committee, provided steps were taken to protect the anonymity of Twitter members. In line with wider ethical guidance [41], we collected publicly posted tweets, avoided verbatim quotations of tweets and have omitted member names in reporting (with the exception of public figures).

## 3 Results

### 3.1 Member characteristics

Following data collection, a total of 189,658 tweets were retained that met the inclusion criteria. Tweets were generated by 109,171 unique members, 17.7% (N = 19,336) of whom tweeted more than once. Half (44.6%) of all members could be matched to a country using the self-reported location field Overall, we matched 31.0% of all members to locations in the USA and 3.3% to locations in the UK (see Table 1).

We were able to generate bot scores for 66.8% (N = 75,116) of the sample overall, including 77.1% (N = 2808) of UK members and 72.2% (N = 24,432) of USA members. A Mann–Whitney U test showed that there was a small, though significant, difference between the median bot scores for UK and USA members (0.13 vs. 0.10; W = 162 288 278.5, *P* < .001) (see Table 2).

**Table 1:** Tweet and unique member counts by memberlocation^a^.

Member location*	Tweets		Members		Tweets per member - tweeted once	
	N	%	N	%	N	%
UK	8952	4.7	3641	3.3	2779	76.3
USA	61,668	32.5	33,836	31.0	27,158	80.3
Other country	25,126	13.2	10,757	9.9	8562	79.6
Not known	95,247	50.2	61,553	56.4	51,785	84.1
All	189,658	100	109,171	100	89,835	82.3

a Note that members can be allocated to more than one location, so may be counted more than once.

### 3.2 Word frequency

We ranked the 50 most frequently used words in the corpus overall, and for UK and USA members (see Supplemental Table 1). As expected, several of the most frequently used terms overall were also keywords included in the list of keywords used to query the API (e.g. ‘vape’ and ‘ecig’). Excluding those keywords included in the search query, the next most commonly used word was ‘smoke’, which appeared 22,601 times, followed by ‘just’ (N = 16,171), ‘like’ (N = 15,070) and ‘get’ (N = 13,615).

We also used a Sankey plot to visualise the overlap between the 25 most frequently used words used by USA and UK members (Supplemental Figure 1). To improve presentation, we excluded the word ‘vape’ from the plot, which was disproportionately prevalent in both countries (see Supplemental Table 1). This comparison showed that words referring to vaping-related health and policy (e.g.‘realdonaldtrump’, ‘ban’ and ‘lung’) ranked more highly in the USA than the UK. Words used as hashtags favoured by the vaping community (e.g. ‘vapefam’ and ‘vapeon’) were more influential in UK tweets. Terms common to both countries included nouns related to e-cigarette products and features (e.g. ‘juul’ and ‘flavour’) as well as verbs indicating intended uses (e.g. ‘smoke’, ‘get’ and ‘use’).

### 3.3 Tweet topics

After training LDA models with different parameters and plotting coherence (supplemental Figure 2), we ultimately selected a topic model with ten topics ($C_{V}=0.46$). This was visualised using an intertopic distance map to show the relationships between topics and counts of words included in topic word sets (see supplemental Figure 3).

Following manual review of tweets assigned to each topic (on the basis of dominant topic contribution), we grouped the topics into three broad themes, as follows (Table 2): informal vaping discussion (topics 1, 2 and 5); vaping policy discussion and EVALI news (topics 3, 4, 7 and 8); and vaping commerce (topics 6, 9 and 10).

Theme 1 **Informal Vaping Discussion**

**Fig. 2:**
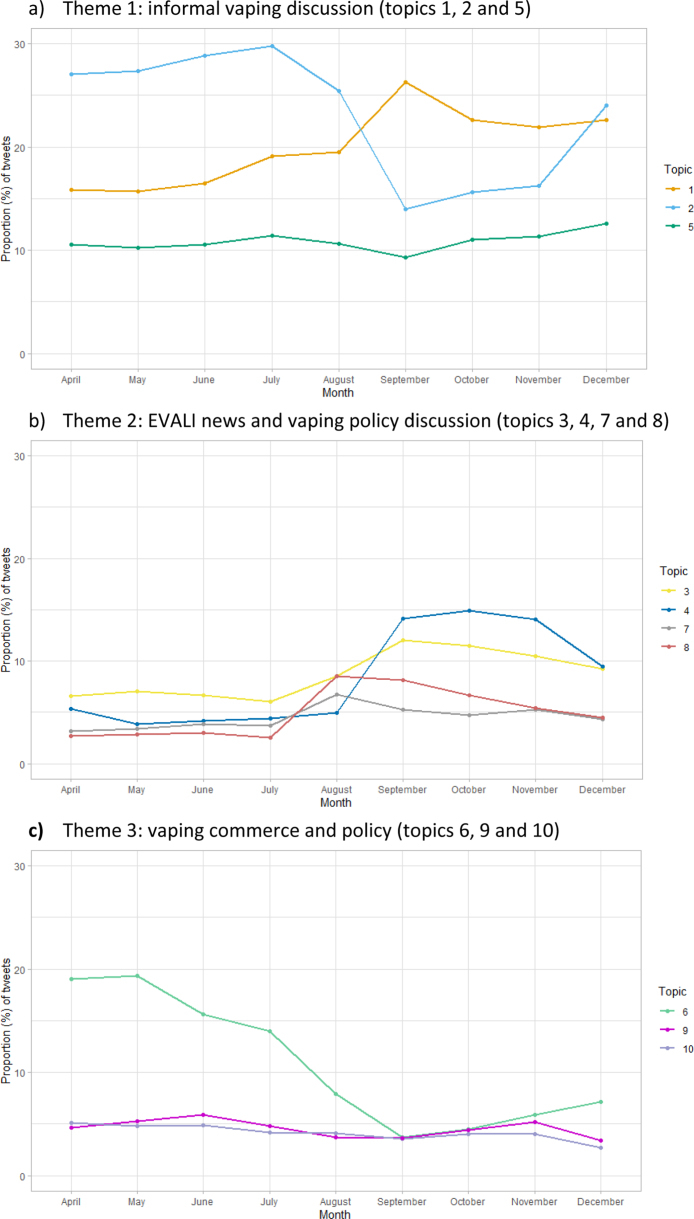
Proportion (%) of tweets allocated to dominant tweet topics over time, by theme and topic.

Theme one comprised the three largest topics. These were closely related when mapped, showed clear semantic similarities in topic content and collectively accounted for over half of the corpus (supplemental Figure 3). Tweets discussed reasons for vaping (or not), anecdotes and experiences, and preferred brands. The language used was often informal, non-standard (e.g. ‘y’all’, ‘hit that vape’) and occasionally profane (see word set, Table 2). Topic 2 contained the majority of references to the brand JUUL. None of the word sets for these three topics referenced cannabinoid products. Bot scores for the three topics in this theme were ranked the lowest among the corpus overall, indicating lower levels of bot activity. Notably, the proportion of tweets assigned to topic 2 showed a sharp decrease in August at the peak of the EVALI crisis (Fig. 2).

Theme 2 **Vaping Policy Discussion and EVALI News**

Tweets in this theme included news reports about the EVALI outbreak including reports of deaths and speculation about possible causes, lobbying and debate about the relative risks of vaping and smoking. References to THC appeared in the word sets for topics 3 and 8; upon inspection, tweets that mentioned THC were commonly directed at distinguishing illegal and/or blackmarket products from regulated, licit, nicotine-based products. In particular, tweets in topic 4 included references to and commentary directed at political figures (‘realdonaldtrump’, N = 4151), policy-oriented discussion (‘ban’, N = 3914), and hashtags aligned with pro and anti-vaping lobbyist communities (#wevapewevote, N = 1845; #parentsvsvaping, N = 367). References to potentially harmful impacts on children and young people were particularly prevalent in topic 3 (‘youth’, N = 1391; ‘teen’, N = 1281). These included fears and counter-arguments addressing underage access to vaping products (regulated and unregulated), the prevalence of vaping among young people and the risk of ENDS use leading to tobacco smoking (the gateway hypothesis). The proportion of tweets assigned to topic 3 showed an increase over time as the EVALI crisis developed (Fig. 2).

Theme 3 **Vaping Commerce**

Topics assigned to theme three mainly comprised posts aimed at selling vaping products, discussing vape shops and reporting industry news. Bot scores for the three topics in this theme were ranked the most highly among the corpus overall (Table 2). Topic 6 was most clearly focused on advertising vaping products and yielded both the highest topic coherence score and bot score for any topic (Table 2). The proportion of tweets assigned to topic 6 showed a clear decrease at the peak of the EVALI crisis (Fig. 2). Tweets in topics 9 and 10 showed some semantic similarities with topics in theme 2, owing to overlapping mentions of cannabinoid-based products. However, as topics 9 and 10 were more skewed towards advertising, we ultimately categorised these under theme three. Topics 9 and 10 also yielded relatively low topic coherence scores adding further confirmation of their heterogeneity.

#### 3.3.1 International differences

**Fig. 3:**
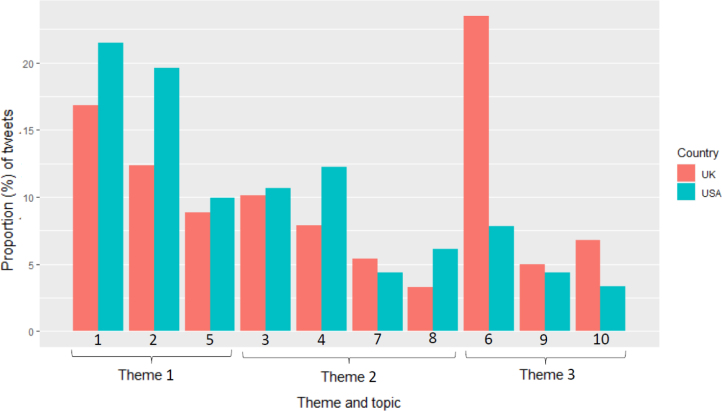
Prevalence (%) of tweets by UK and USA members assigned to LDA-generated topics, by country. Legend: Topics are ordered firstly by theme and then by topic number.

Among the subset of tweets by members in the UK and USA, we compared the prevalence of each topic (Fig. 3). The most prominent difference was the marked dominance of commerce-related (topic 6) tweets in the UK (23.5% vs. 7.8%; x2 = 2165.0, df = 1, *P* < .001). This was the most common topic assigned to UK tweets, though only the sixth most common among USA tweets (Fig. 3).

**Table 2:** Tweet topics yielded using optimal LDA model (10 topics), by theme .

Topic number^a^ and description, by theme		Word set^b^ (N = 10)	Topic size^c^, % (N)	Topic coherence (Cv score)	Bot score (median, IQR)
Theme 1: Informal vaping discussion					

1	Informal discussion about reasons for and against vaping, including comparisons with smoking.	Smoke, peopl, don’t, like, cigarett, juul, know, kid, get, would	20.5 (38,838)	0.50	0.11 (0.18)

2	Informal vaping-related discussion, experiences and anecdotes. Mainly positive sentiment with brand- specific references.	juul, hit, I’m, juul_pod, like, got, fuck, pen, get, one	23.1 (43,798)	0.42	0.07 (0.15)

5	Informal vaping social commentary. Mixed sentiment. Topics include vaping in public, celebrities vaping and brand-specific comments.	juul, get, don’t, need, y’all, man, want, like, go, know	11.3 (21,422)	0.40	0.12 (0.23)

Theme 2: Vaping policy discussion and EVALI news					

3	Debate on the health effects of vaping (inc. illicit EVALI and THC products) and role as a harm reduction tool, with references to concerns about harms to children and young people.	Use, nicotin, product, ecig, ecigarett, tobacco, youth, market, THC, flavor	8.8 (16,676)	0.51	0.16 (0.25)

4	Politically orientated commentary, lobbying and discussion about vaping policy, including proposed bans and/or restrictions on vaping products e.g. flavoured e-liquids. References to US political figures.	Ban, realdonaldtrump, flavour, product, FDA, wevapewevot, industri, thank, issu, vaper	8.9 (16,847)	0.57	0.15 (0.24)

7	News and commentary on EVALI, the health effects of vaping and other adverse events (e.g. explosions), inc. comparisons with smoking tobacco.	Smoke, cigarett, lung, caus, tobacco, year, health, doctor, ecig, studi	4.4 (8429)	0.46	0.17 (0.25)

8	News and commentary about EVALI related illnesses and deaths and the associated investigations.	Report, ill, case, link, death, US, THC, state, lifestyl, CDC	4.7 (8822)	0.44	0.19 (0.35)

Theme 3: Vaping commerce					

6	Advertising for vaping related products, liquids and kits.	New, eliquid, vapour, vaper, kit, cartridg, giveaway, ecig, pod, mod	10.0 (19,045)	0.63	0.40 (0.40)

9	News and commentary relevant to the vaping commercial industry, including news of bans and restrictions on vaping products and vape shops (inc CBD and cannabis related). Some product advertising.	Sale, flavor, ban, CBD, cannabi, store, juic, shop, product, featur	4.4 (8415)	0.36	0.23 (0.41)

10	Advertising for edible CBD and products. Also some mention of EVALI reports among students.	Ecigarett, via, new, total_hit, custom_view, compani, student, rip, cbdcandi_buycbd, cbdstore_cbdedibl	3.9 (7366)	0.39	0.31 (0.35)

a Topics may be numbered out of sequence in order to match the automated numbering system used in the intertopic distance visualisation output.

b Words presented in their stemmed forms, if applicable, underscore (_) indicates bigram. Word set includes most frequently occurring 10 words per topic (ranked most frequent to least frequent).

c Number of tweets where the topic has been allocated as the dominant topic for that tweet.

## 4 Discussion

### 4.1 Principal results

To the best of our knowledge, this is the first study to use topic modelling to analyse social media commentary about ENDS during the EVALI outbreak. The topics we found and patterns over time observed suggested that the EVALI outbreak in the USA disrupted usual social media commentary about ENDS by prompting a wave of news stories and discussion on Twitter internationally about the resulting illnesses and deaths, and speculation on the causes. There were also signs of increased discussion about not only vaping products containing THC, but topics about the safety of ENDS more broadly including vaping policy and regulation (e.g. restrictions on permitted flavourings) and the relative health risks and benefits of ENDS compared to smoking tobacco. Analysis of international differences suggested such topics were not confined to USA members. Throughout this period, we also found a relatively stronger presence of commercially orientated tweets and automated bot accounts focused on marketing ENDS products among UK members compared to USA members.

### 4.2 Comparison with prior work

Previous studies about ENDS have highlighted the dominance of commercial advertising and informal social commentary about vaping community and culture [21], [24], [42]. While informal discussions about ENDS remained the most prevalent topics of conversation among Twitter members in our study period (theme 1), matters of health and policy were also relatively prevalent (theme 2). Our findings align with and support previous studies. A previous study that analysed news articles found that stories about the dangers of vaping hit record highs following EVALI [43]. Another study analysing themes among tweets including the phrase ‘flavours save lives’ following the EVALI outbreak noted unsubstantiated health claims, particularly the belief that flavours aided smoking cessation [44].

The findings of our study, in the wider context of related work, indicate that the EVALI outbreak – while primarily linked to THC products – may have, at least temporarily, further amplified and disrupted health-related discourses online about ENDS generally in potentially important ways. How the EVALI outbreak and the subsequent media coverage that followed may have affected public understanding of risk in relation to ENDS is not yet fully understood. However, both UK and USA surveys have noted increases in the proportion of adults who perceived ENDS to be as harmful as or more harmful than cigarettes [45], [46], suggesting the need for clearer communications about the relative and absolute harms of ENDS. Such communications should arguably target social media platforms, particularly to counter misinformation, unsubstantiated health claims or other information disseminated to selectively perpetuate particular ideas about ENDS [42].

We found that vaping commerce tweets accounted for a higher proportion of tweets in the UK than in the USA. This likely reflects the UK’s established public health position, which accepts ENDS as part of smoking cessation strategies. Indeed, there were only two suspected EVALI cases in the UK [15] - products containing THC or vitamin E acetate are not available on the licit UK market [18]- so it is plausible that UK retailers of nicotine based ENDS may have been less affected. Other possible explanations may be that USA ENDS retailers consciously kept lower profiles during the EVALI outbreak. It is notable that JUUL voluntarily suspended sale of fruit flavoured products in October 2019, ahead of a broader countrywide ban on certain flavoured products in January 2020 [47]. One study reported a significant contraction in online shopping queries for vaping products, including JUUL specifically, during the outbreak [48]. Thus, it seems possible that online commerce via social media was also disrupted during EVALI. Nonetheless, without examining the overall prevalence of tweets, which the API did not allow, we cannot conclusively determine the reason.

### 4.3 Limitations

Though a large dataset, our sample was limited to public tweets in English on a single social media platform. As the topics derived were shaped by our keyword search strategy, we may have omitted important keywords and brands. Furthermore, the inclusion of particular keywords (‘JUUL’ in particular) may have resulted in USA tweets being over-represented. More sophisticated techniques to define and expand keywords (e.g. use of word embeddings) may have captured a more representative set of tweets. Though we deemed the topics yielded by our optimum model to be sufficiently interpretable and coherent, evaluating topic models is a complex task, complicated further by the brevity of Twitter posts. Future work could incorporate experimental approaches, alongside statistical measures, to validate topics [49]. Furthermore, the volume of ENDS related tweets and constraints of sampling via the ‘black box’ of Twitter’s API means we could not obtain all relevant tweets nor guarantee a truly random sample.

Limited meta-data about members meant we had rely on algorithms to infer their characteristics and thus generate key variables (e.g. location and bot scores). Locations could not be derived for a significant proportion of members. Furthermore, locations that could be derived were based on algorithmic assessments of member-generated, self-report data (free text). First, it is likely that a small proportion could have been inaccurate or out of date. Second, even if accurately recorded by the user, our brief testing of the accuracy indicated that USA locations were likely under detected by our combination of algorithmic methods. As only tweets in English were included, it is likely that people of White ethnic origin were overrepresented for tweets in both countries. Indeed, previous studies have reported complex, and sometimes subtle, differences in ENDS perceptions, intentions and use among people of different ethnicities [50], [51]. Though we explored the relative presence of bot-like users among tweet topics, we did not exclude bots meaning the topics derived may not wholly represent those discussed by genuine Twitter users. Our decision to exclude duplicate tweets may have attenuated the influence of more prolific bots. Though several of these limitations arguably reflect the well-known complexities of working with social media data and are not unique to our study [52], they do warrant caution in interpreting our results and limit generalising to more diverse populations.

## 5 Conclusions

This study has identified and described how online Twitter discussions during the EVALI outbreak affected general commentary about ENDS and how topics changed over time as the crisis unfolded. The study also identified notable differences in content between people who use Twitter in the USA and the UK, where vaping policy approaches also differ. Furthermore, we have contributed to a growing evidence base demonstrating the relevance of using large-scale, social media data to yield insights relevant to understanding (and shaping) public discourses relevant to tobacco control policy and issues [20], [21], [24], [44]. Future research about attitudes towards ENDS may benefit from triangulating social media data with more formal sources, such as longitudinal epidemiological studies or qualitative research data where available.

## Declaration of Competing Interest

The authors declare that they have no known competing financial interests or personal relationships that could have appeared to influence the work reported in this paper.

## Appendix A Supplementary data

MMC S1
