# Supplementary material for: Text mining tweets on e-cigarette risks and benefits using machine learning following a vaping related lung injury outbreak in the USA
Source: Healthc Anal (N Y). 2022 Nov;2:None. doi: 10.1016/j.health.2022.100066 (PMC9801957; doi:10.1016/j.health.2022.100066)

**SUPPLEMENTAL MATERIALS**

**Supplemental Table 1** – Top 50 words, based on overall word frequency within the corpus

| UK | | | | | | USA | | | | | | ALL | | | | | |
| --- | --- | --- | --- | --- | --- | --- | --- | --- | --- | --- | --- | --- | --- | --- | --- | --- | --- |
| Rank | **Word** | **N** | **Rank (ctd.)** | **Word** | **N** | **Rank** | **Word** | **N** | **Rank (ctd.)** | **Word** | **N** | **Rank** | **Word** | **N** | **Rank (ctd.)** | **Word** | **N** |
| 1 | vape | 8509 | **26** | vapeshop | 429 | **1** | vape | 51339 | **26** | e-cigarett | 2785 | **1** | vape | 150004 | **26** | want | 7052 |
| 2 | smoke | 1203 | **27** | peopl | 426 | **2** | juul | 12683 | **27** | quit | 2775 | **2** | juul | 40067 | **27** | lung | 7043 |
| 3 | new | 1110 | **28** | review | 426 | **3** | smoke | 7386 | **28** | thc | 2760 | **3** | smoke | 22601 | **28** | year | 6787 |
| 4 | vapefam | 1097 | **29** | tobacco | 418 | **4** | just | 5174 | **29** | kid | 2758 | **4** | just | 16171 | **29** | make | 6777 |
| 5 | ecig | 1023 | **30** | product | 413 | **5** | flavour | 5084 | **30** | say | 2712 | **5** | like | 15070 | **30** | quit | 6691 |
| 6 | vapeon | 841 | **31** | ublo | 408 | **6** | get | 5052 | **31** | make | 2487 | **6** | get | 13615 | **31** | cbd | 6652 |
| 7 | vapecommun | 790 | **32** | ban | 407 | **7** | like | 4945 | **32** | want | 2423 | **7** | use | 11559 | **32** | e-cigarett | 6624 |
| 8 | vaper | 753 | **33** | health | 402 | **8** | product | 4863 | **33** | cbd | 2346 | **8** | new | 11252 | **33** | health | 6443 |
| 9 | flavour | 712 | **34** | us | 394 | **9** | ban | 4687 | **34** | need | 2275 | **9** | peopl | 11120 | **34** | kid | 6273 |
| 10 | now | 696 | **35** | go | 387 | **10** | use | 4553 | **35** | time | 2206 | **10** | can | 10945 | **35** | need | 6207 |
| 11 | eliquid | 680 | **36** | shop | 382 | **11** | peopl | 4213 | **36** | teen | 2199 | **11** | ban | 10637 | **36** | time | 5930 |
| 12 | vapelif | 671 | **37** | ejuic | 376 | **12** | nicotin | 4077 | **37** | ecig | 2187 | **12** | now | 10479 | **37** | think | 5857 |
| 13 | get | 669 | **38** | liquid | 371 | **13** | can | 3740 | **38** | shop | 2122 | **13** | nicotin | 10372 | **38** | thc | 5596 |
| 14 | cbd | 588 | **39** | year | 343 | **14** | tobacco | 3676 | **39** | think | 2046 | **14** | pod | 10328 | **39** | stop | 5526 |
| 15 | vapingtrain | 582 | **40** | pod | 331 | **15** | cigarett | 3667 | **40** | us | 2038 | **15** | product | 10053 | **40** | tri | 5481 |
| 16 | use | 580 | **41** | day | 328 | **16** | realdonaldtrump | 3607 | **41** | tri | 2027 | **16** | flavour | 9718 | **41** | pen | 5351 |
| 17 | like | 560 | **42** | e-liquid | 324 | **17** | now | 3563 | **42** | vapour | 2000 | **17** | cigarett | 9185 | **42** | us | 5285 |
| 18 | e-cigarett | 540 | **43** | need | 321 | **18** | one | 3191 | **43** | stop | 1992 | **18** | one | 9071 | **43** | vapour | 5257 |
| 19 | just | 525 | **44** | make | 314 | **19** | lung | 3039 | **44** | market | 1964 | **19** | tobacco | 8721 | **44** | day | 5153 |
| 20 | nicotin | 503 | **45** | time | 311 | **20** | pod | 3020 | **45** | vaper | 1941 | **20** | ecig | 7945 | **45** | look | 4809 |
| 21 | juul | 490 | **46** | anon* | 309 | **21** | health | 2874 | **46** | hit | 1933 | **21** | know | 7874 | **46** | take | 4800 |
| 22 | uk | 481 | **47** | tri | 307 | **22** | new | 2862 | **47** | day | 1898 | **22** | go | 7790 | **47** | juic | 4693 |
| 23 | one | 470 | **48** | vapen | 302 | **23** | know | 2861 | **48** | pen | 1879 | **23** | hit | 7664 | **48** | see | 4657 |
| 24 | kit | 438 | **49** | know | 295 | **24** | go | 2818 | **49** | cannabi | 1878 | **24** | say | 7131 | **49** | got | 4646 |
| 25 | can | 431 | **50** | smoker | 293 | 25 | year | 2787 | **50** | ill | 1826 | **25** | vaper | 7104 | **50** | buy | 4636 |

*Individual username (i.e. non-brand and not a public figure) removed to preserve anonymity

**Supplemental Figure 1:** Sankey plot of the most frequently used 25 words in e-cigarette tweets by users in a) the UK and b) USA

REVIEWERS - Note this is a screen shot and the actual file is interactive (html multimedia file)


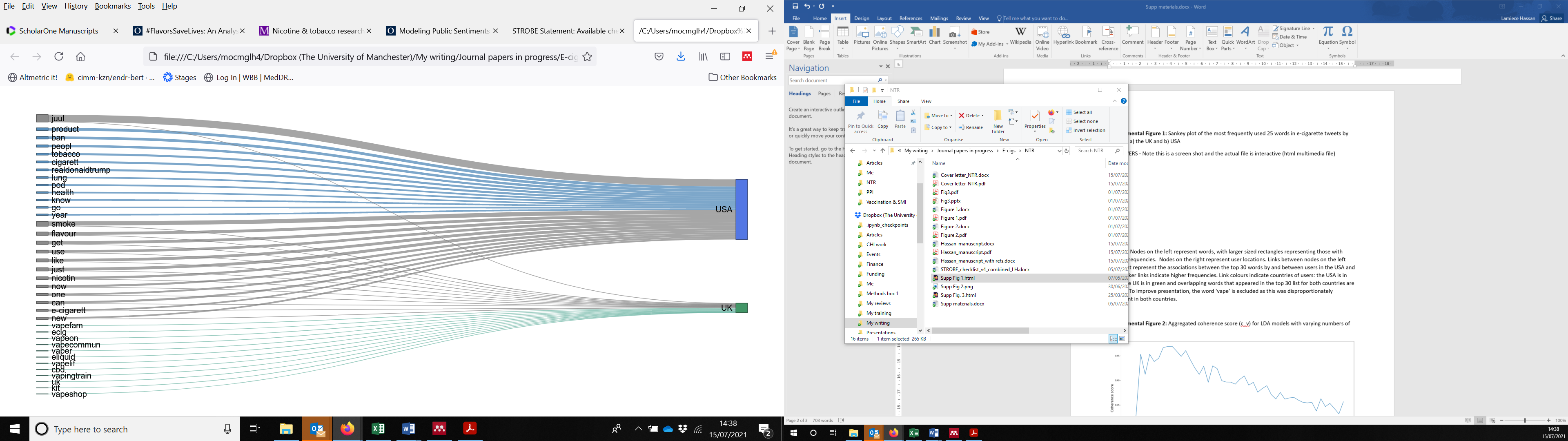


Legend: Nodes on the left represent words, with larger sized rectangles representing those with higher frequencies. Nodes on the right represent user locations. Links between nodes on the left and right represent the associations between the top 30 words by and between users in the USA and UK: thicker links indicate higher frequencies. Link colours indicate countries of users: the USA is in blue, the UK is in green and overlapping words that appeared in the top 30 list for both countries are in grey. To improve presentation, the word ‘vape’ is excluded as this was disproportionately prevalent in both countries.

**Supplemental Figure 2:** Aggregated coherence score (c_v) for LDA models with varying numbers of topics

**
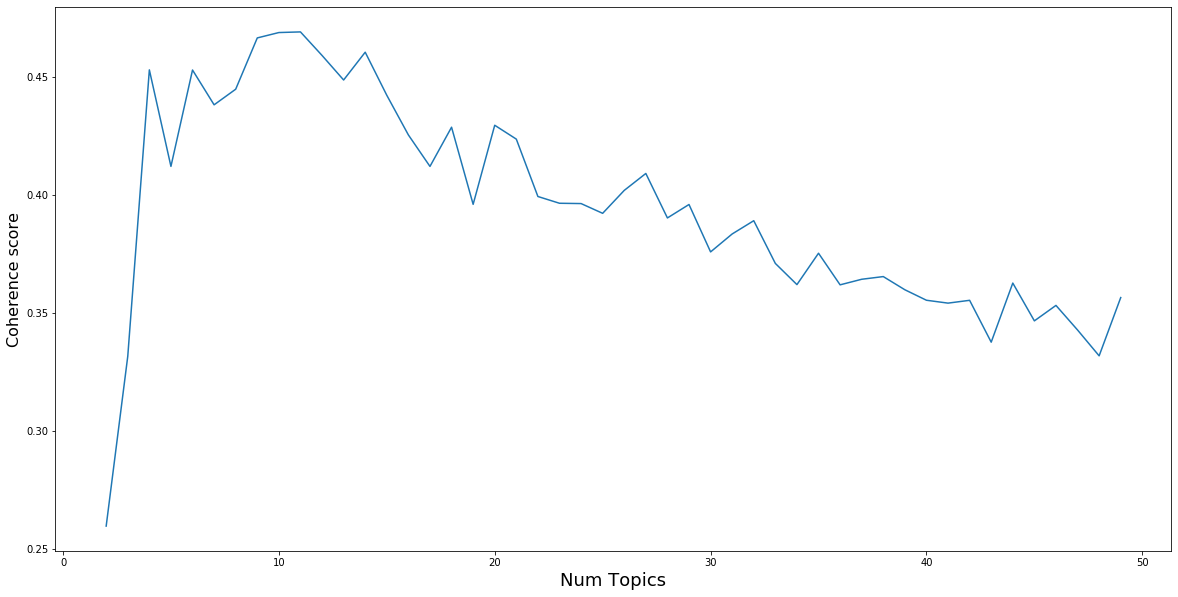
**

**Supplemental Figure 3:** Intertopic distance map for optimal LDA model with 10 topics

REVIEWERS - Note this is a screen shot and the actual file is interactive (html multimedia file)

Legend: Topics are represented as bubbles of varying size. Proximity/overlap indicates topic similarities.


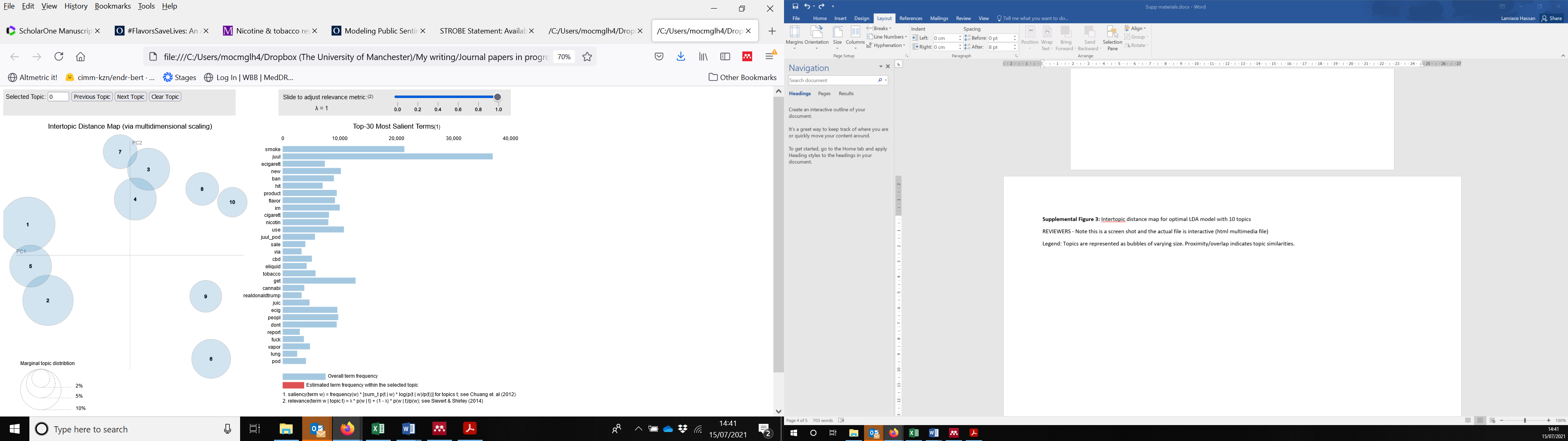

Supplement: MMC S1 [file mmc1.zip › mmc1/mmc1.docx]
