# Supplementary figures and images for: Text mining tweets on e-cigarette risks and benefits using machine learning following a vaping related lung injury outbreak in the USA
Source: Healthc Anal (N Y). 2022 Nov;2:None. doi: 10.1016/j.health.2022.100066 (PMC9801957; doi:10.1016/j.health.2022.100066)

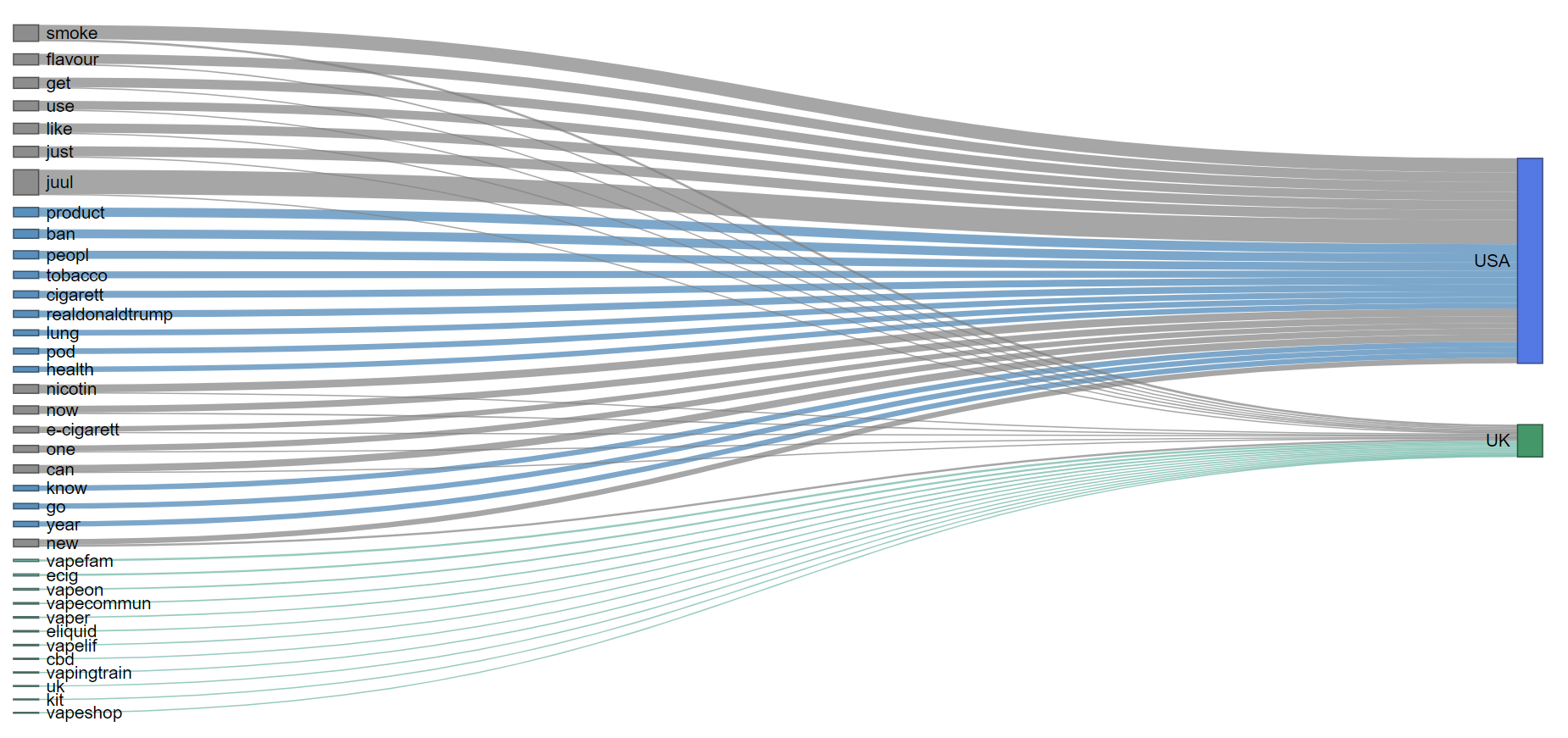

Supplement: MMC S1 [file mmc1.zip › mmc1/Supplementary Figure 1.PNG]

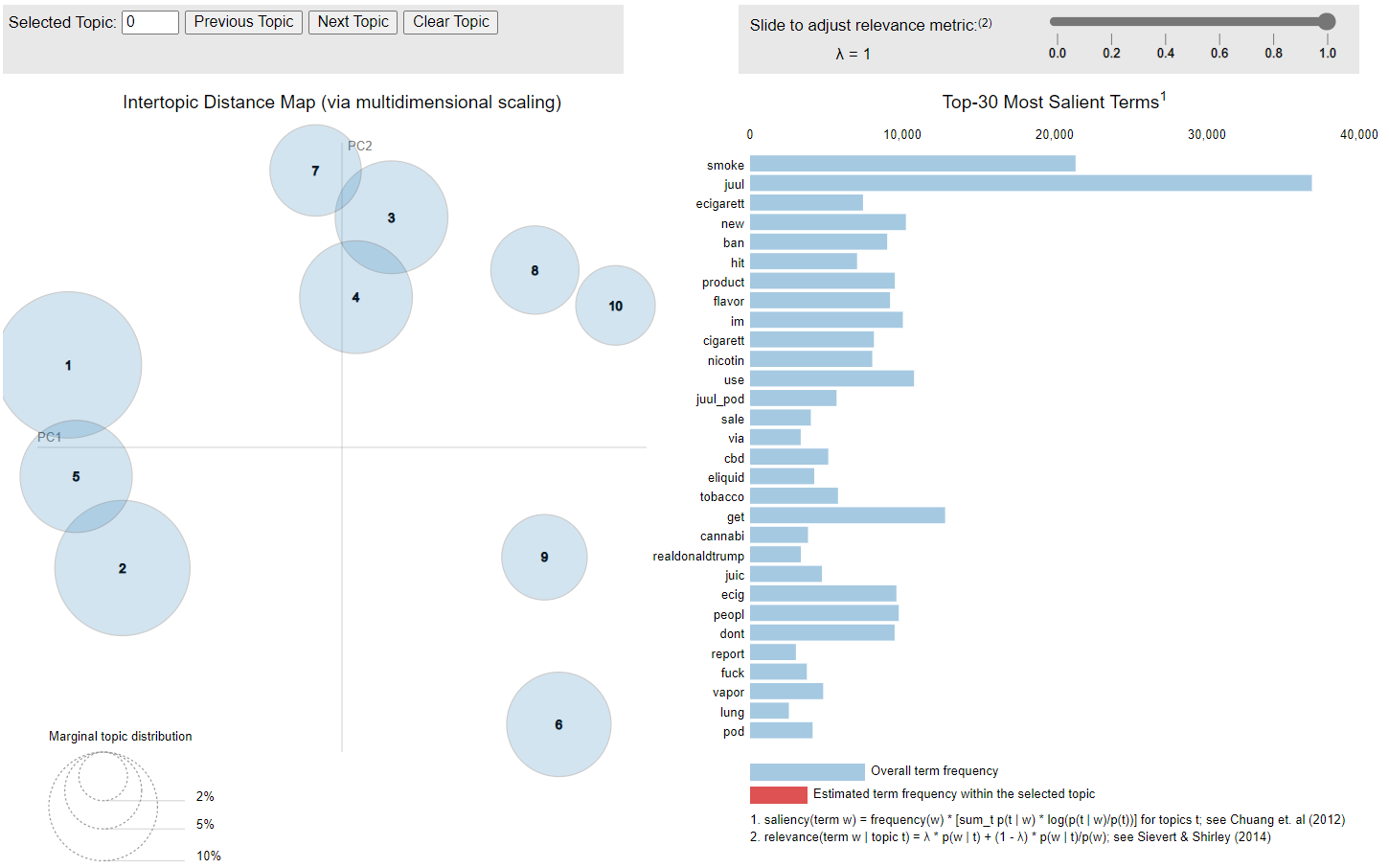

Supplement: MMC S1 [file mmc1.zip › mmc1/Supplementary Figure 2.PNG]
